# Burden of Injuries in Bangladesh: A Population-Based Assessment

**DOI:** 10.3390/ijerph15030409

**Published:** 2018-02-27

**Authors:** Priyanka Agrawal, Adnan A. Hyder

**Affiliations:** Department of International Health, International Injury Research Unit, Johns Hopkins University Bloomberg School of Public Health, Baltimore, MD 21205, USA; pagrawa6@jhu.edu

**Keywords:** injury, population-based, survey, drowning, epidemiology, Bangladesh

Injuries claim over 5 million lives, with more than 90% of those occurring in low- and middle-income countries (LMICS) [1,2]. Unintentional injuries such as drowning, road traffic injuries, falls and burns account for 72% of all injury deaths. Drowning, one of the leading causes of unintentional injuries across the world, accounted for more than 300,000 deaths in 2016 [3]. This collection documents some of the epidemiological findings on the burden of injuries, both intentional and unintentional, in Bangladesh, in the context of a large, multi-year, population-based project—Saving of Lives from Drowning (SoLiD).

SoLiD was established with the main objective of evaluating the large-scale effectiveness and value for money of interventions—crèches and playpens—in the reduction of drowning mortality and morbidity in children less than 5 years of age. The project conducted a baseline census in seven rural sub-districts of Bangladesh and covered over 1.2 million individuals, in order to collect demographic and injury-related information. An injury surveillance system was set up to collect injury outcomes on a quarterly basis for 3 years, and compliance assessments on a monthly basis to test the usability and acceptability of the interventions.

This collection highlights the epidemiology and risk factors for injuries prevalent in rural Bangladesh, and showcases the depth of information generated from a large population-based survey. While the collection provides a snapshot of the burden of injury in a low- and middle-income country such as Bangladesh, it also highlights the slow progress, via the dearth of available evidence-based effective interventions, programs and policies present to address this burden.

In Bangladesh, while childhood deaths due to communicable infectious diseases were on a decline in the past decade, deaths due to injuries in the same age group were increasing. The paper *Epidemiology of Drowning in Bangladesh: An Update* shows that children 1 to 5 years of age were 13 to 16 times more likely to be involved in a drowning (or near-drowning) event than infants or older children. Individuals from lower socio-economic profiles were at more risk of drowning than their rich counterparts. Males also sustained more near-drowning events than females.

A similar gender trend is highlighted in the paper on the *Pattern of Road Traffic Injuries in Rural Bangladesh: Burden Estimates and Risk Factors*. The authors suggest developing policies and programs to make pedestrian-friendly road networks and reinforcement of helmet use, given that pedestrians and two-wheel drivers sustained more than one-third of road traffic injuries.

In contrast, burn injuries and suicides were three to six times more common in females than in males in the rural population of Bangladesh. In *Epidemiology of Burns in Rural Bangladesh: An Update*, the authors also overcame the issue of under-reporting and underestimation of the burden of burn injuries in a LMIC setting. In *The Burden of Suicide in Rural Bangladesh: Magnitude and Risk Factors*, adolescent girls and young married women, aged 15 to 24 years of age, showcased a very specific age group at a disproportionately high risk.

Additionally, in *Epidemiology of Fall Injury in Rural Bangladesh*, elderly people, 65 years of age and above, were seen to be at highest risk of both fatal and non-fatal fall injuries. The physical, mental and emotional wellbeing of elderly individuals can be disrupted by a debilitating fall injury and drive them into greater vulnerability. These papers highlight the need for targeted approaches in the form of interventions, policy changes and programs to address respective burdens of disease.

The notion of a center-based, early childhood education program may not be entirely new in Bangladesh, but there is a lack of research on the cognitive benefits of such an approach in LMICs. In high-income countries, it has been shown that early childhood education has both short term and long-term benefits for children exposed to it. The paper *Developmental Assessments during Injury Research: Is Enrollment of Very Young Children in Crèches Associated with Better Scores?* showed that being enrolled in a crèche intervention had a positive dose-response relationship on fine and gross motor skills, personal–social and problem-solving skills for children 1–5 years of age. This reiterated the importance of engaging young children in formal age-appropriate education systems.

The authors of *Caregiver Supervision Practices and Risk of Childhood Unintentional Injury Mortality in Bangladesh*, used a large population-level dataset to suggest that adult caregiver supervision significantly reduced the risk of drowning deaths for children under 5 years of age. This notion of adult supervision needs to be effectively communicated amongst communities in LMICs, mainly because injuries are still conceived of as unforeseeable “accidents” that cannot be prevented. Additionally, with injuries comes a high risk of sustaining a long-term disability, along with loss of productivity and other economic costs. First aid, by a formal medical provider, can improve the odds of faster recovery for an individual with injuries. However, informal providers are readily available in LMICs such as Bangladesh. Thus, authors of the paper *Impact of First Aid on Treatment Outcomes for Non-Fatal Injuries in Rural Bangladesh: Findings from an Injury and Demographic Census*, suggest that public health interventions should be designed to train and improve first aid skills for informal providers.

It is important to consider the economic ramifications of injuries and associated disabilities, because the majority one-income households in rural Bangladesh undergo financial distress in dealing with the aftermath of fatal and non-fatal injuries. In the paper, *Care-Seeking Patterns and Direct Economic Burden of Injuries in Bangladesh*, the authors tested the relationship between care-seeking behavior of injured households and financial distress, and suggested that enforcing occupational safety regulations, worksite inspections, home safety inspections and such promotions, should help alleviate the economic distress caused by injury.

This collection of papers, therefore, provides a base for new channels of future research. These scholars have shown correlations between injury and age, sex and wealth. Additionally, they focus on demonstrating the link between various types of interventions—be it childcare centers, playpens, or first aid training—and injury prevention. What needs further study is *why* certain correlations exist and the *how* specific interventions work. To address these deficiencies, future studies must be of longer duration to include the eventual impact and include qualitative measurements of behavior to trace links between risks and actual injury prevention. The group involved in these papers is now working to address this gap in incidence. When researchers have a better handle on causal links, they can also look at antecedents, such as cultural and social norms, that influence behavior, all questions that need to be addressed to reduce the millions of deaths caused by injuries around the world.
